# Atorvastatina Reduz o Acúmulo de Células Musculares Lisas Vasculares para Inibir a Hiperplasia Intimal pela Inibição de Via p38 MAPK em um Modelo de Enxerto de Veia em Ratos

**DOI:** 10.36660/abc.20190231

**Published:** 2020-10-13

**Authors:** Tianshu Chu, Molin Huang, Zhiwei Zhao, Fei Ling, Jing Cao, Jianjun Ge

**Affiliations:** 1 University of Science and Technology of China Division of Life Sciences and Medicine The First Affiliated Hospital of USTC Hefei China Department of cardiac Surgery, The First Affiliated Hospital of USTC, Division of Life Sciences and Medicine, University of Science and Technology of China, Hefei – China

**Keywords:** Atorvastatina, Miócitos de Músculo Liso, Hiperplasia, Modelos Animais, Quinases de Proteína Quinase Ativadas por Mitógenos, Revascularização do Miocárdio, Ratos

## Abstract

**Fundamento:**

A taxa de falha de enxerto de veia safena um ano após a cirurgia de revascularização do miocárdio varia de 10% a 25%. O objetivo deste estudo foi de investigar se a atorvastatina pode reduzir o acúmulo de células musculares lisas vasculares para inibir a hiperplasia intimal por meio da inibição da via p38 MAPK.

**Métodos:**

Quarenta e cinco ratos Sprague-Dawley foram randomizados em três grupos. Trinta ratos foram submetidos à cirurgia de enxerto de veia e randomizados para tratamento com veículo ou atorvastatina; quinze ratos foram submetidos à cirurgia sham. Detectamos a hiperplasia intimal por meio de coloração com hematoxilina-eosina e a expressão de proteínas relacionadas por meio de análise imuno-histoquímica e Western blot. Foram realizadas as comparações por análise de variância de fator único e pelo teste da diferença mínima significativa de Fisher, com p < 0,05 considerado significativo.

**Resultados:**

A íntima analisada pela coloração com hematoxilina-eosina era dramaticamente mais espessa no grupo controle que no grupo atorvastatina e no grupo sham (p < 0,01). Os resultados da coloração imuno-histoquímica de α-SMA demonstraram que a porcentagem de células positivas para α-SMA no grupo controle era mais alta que no grupo atorvastatina (p < 0,01). Nós também avaliamos α-SMA, PCNA, p38 MAPK e fosforilação de p38 MAPK após o tratamento com estatina por meio de análise de Western blot e os resultados indicaram que a atorvastatina não levou à redução de p38 MAPK (p < 0,05); no entanto, resultou na inibição da fosforilação de p38 MAPK (p < 0,01) e reduziu significativamente os níveis de α-SMA e PCNA, em comparação com o grupo controle (p < 0,01).

**Conclusão:**

Nós demonstramos que a atorvastatina pode inibir o acúmulo de células musculares lisas vasculares por meio da inibição da via p38 MAPK e é capaz de inibir a hiperplasia intimal em modelos de enxerto de veia em ratos.

## Introdução

A doença arterial coronariana e as complicações relacionadas ainda são as principais causas de mortalidade em todo o mundo, embora tenha havido muitos avanços na terapia médica. Diversos estudos e diretrizes clínicas mostram que a cirurgia de revascularização do miocárdio (CRM) reduz a morbimortalidade de pacientes com doença triarterial ou doença principal esquerda, com fração de ejeção reduzida em comparação com a intervenção coronária percutânea.^1^ Notavelmente, um ano após a CRM, a taxa de falha do enxerto de veia safena (EVS) pode ser de 10% a 25%; no período de 1 a 5 anos, a taxa aumentará de 1% a 2% por ano.^2^^,^^3^ Além disso, em 6 a 10 anos, a taxa de bloqueio aumenta de 4% a 5% por ano, devido à aterosclerose.^4^ O mecanismo da reestenose de EVS inclui trombose, hiperplasia intimal (HI) e aterosclerose. A proliferação e a migração das células endoteliais e das células musculares lisas vasculares (CMLVs) são fundamentais à HI e a HI é a causa principal da reestenose de EVS.^5^ Porém, o mecanismo da HI não é claro e ainda não se sabe qual método de prevenção e terapia seria mais eficaz.

Uma quantidade substancial de evidência sugere que o tratamento com estatina reduza o risco cardiovascular; portanto, quando os níveis de colesterol de lipoproteína de baixa densidade são superiores a 100 mg/dL, a terapia com estatinas é recomendada para os pacientes com doença arterial coronariana.^6^ Muitas observações indicam que o tratamento pré-operatório com estatina pode reduzir a morbidade, a mortalidade pós-operatória e as complicações. Evidências recentes indicam que o tratamento com estatina após CRM pode reduzir a taxa de doença de EVS por meio da inibição de HI, indicando que o tratamento otimizado com estatina é crucial para a avaliação do benefício de longo prazo da CRM.^7^^–^^9^ É amplamente aceita a hipótese de “resposta à injúria” proposta por Russell Ross; esta hipótese afirma que, após a arterialização, o EVS instantaneamente sofre lesões (por exemplo, isquemia, hipóxia, estresse de cisalhamento ou trauma cirúrgico), provocando um evento que inicia a resposta inflamatória, seguida por alterações morfológicas e funcionais levando a HI, como resultado do acúmulo de CMLVs e da disfunção das células endoteliais.^10^ A disfunção, proliferação e migração destas células são estimuladas pela fosforilação de p38 proteínas quinases ativadas por mitógeno (p38 MAPKs), quinases reguladas por sinal extracelular (ERK), c-Jun e quinases N-terminais.^11^ Estudos têm demonstrado que a inibição de p38 MAPK pode reduzir a resposta imune inata e, consequentemente, inibir a HI após a arterialização do EVS.^12^^,^^13^

Com base nesses estudos em HI, formulamos a hipótese de que a atorvastatina poderia reduzir o acúmulo de CMLVs para inibir HI por meio da supressão da via p38 MAPK. Nós testamos a nossa hipótese utilizando um modelo de enxerto de veia com tratamento com estatina em ratos para detectar HI por meio de coloração com hematoxilina-eosina e expressão de proteína correlacionada por meio de análise imuno-histoquímica e de Western blot. Verificamos que a atorvastatina era capaz de inibir a fosforilação de p38 MAPK para reduzir o acúmulo de CMLVs e, adicionalmente, inibir a HI.

## Materiais e Métodos

### Animais Experimentais e Procedimento Cirúrgico

Todos os experimentos com os animais neste estudo foram realizados de acordo com os protocolos aprovados pelo Comitê Institucional para Uso e Cuidado de Animais de Laboratório. Quarenta e cinco ratos machos Sprague-Dawley, de 8 a 10 semanas de idade, livres de patógenos, pesando de 200 a 220 g, foram fornecidos pelo Centro de Pesquisa do Laboratório de Animais de Anhui e identificados pelo Comitê de Ética Médica da Universidade Médica de Anhui. Foram randomizados (desenho completamente randomizado) em 3 grupos, contendo 15 ratos cada, e alimentados durante 4 semanas após a operação. Trinta ratos receberam o enxerto de veia, conforme previamente descrito;^14^ o método foi utilizado para construir modelos de enxerto de veia jugular direita na artéria carótida comum, e os ratos foram randomizados para serem tratados com veículo (grupo controle, administrado com água destilada continuamente por gavagem durante 4 semanas) ou atorvastatina (grupo atorvastatina, 15 mg/kg, dissolvido em água destilada). Quinze ratos receberam uma cirurgia sham (grupo sham), definida como simulação do processo operatório, sem arterialização venosa e intervenção médica.

### Coleção de Amostras

Coletamos o enxerto de veia de cada rato na quarta semana após a operação. Os ratos foram totalmente anestesiados, fixados na mesa de operação, heparinizados como antes e operados da mesma forma, pela mesma abordagem. Para análise histológica, os enxertos venosos foram colocados em microtubos com paraformaldeído e fixados a 4 °C durante 24 horas. Os enxertos venosos com Western Blot foram colocados em microtubos sem solvente e, subsequentemente, armazenados a −80 °C. Os ratos foram sacrificados por deslocamento cervical e devidamente manuseados.

### Análise Histológica e Imuno-histoquímica

Foi realizada a análise morfométrica da íntima por coloração com hematoxilina-eosina, utilizando um kit de coloração de hematoxilina e eosina (Beyotime Biotechnology, Shanghai, China). Foi utilizado um sistema de aquisição de imagem microscópica Olympus para coletar imagens das seções (lentes objetivas ×40, ×100 e ×200) e medir a espessura da íntima. Dois pesquisadores independentes realizaram as medidas e a análise dos dados. Nós selecionamos seções das veias enxertadas; subsequentemente, medimos 16 pontos de espessura intimal e calculamos a média. As seções de tecido foram testados para proliferação celular utilizando um kit para análise imuno-histoquímica para α-actina de músculo liso (α-SMA) (R&D Systems, Bio-Techne, Minnesota, EUA), a proteína específica das CMLVs. Todas as imagens (lentes objetivas ×100 e ×200) foram obtidas utilizado um sistema de aquisição de imagem microscópica Olympus (Olympus, Japan) e processadas com o software Image-J 1.48u (National Institutes of Health, Bethesda, EUA). Um total de 10 observações foram aplicadas para calcular a porcentagem média de células positivas para α-SMA de cada rato.

### Análise de Western Blot

Quatro semanas após a operação, quantidades equivalentes de proteínas dos enxertos venosos dos três grupos foram submetidas à eletroforese em dodecil sulfato de sódio/gel de 10% e transferidas para membranas de PVDF (Sigma-Aldrich, EUA). As membranas foram, subsequentemente, incubadas com anticorpos anti-fosfo-específicos de p38 MAPK, anticorpos anti-não-fosforilados de p38 MAPK, anticorpos anti-não-fosforilados de α-SMA e anticorpos anti-não-fosforilados de antígeno nuclear de proliferação celular (PCNA), seguido por incubação com IgG-peroxidase anti-rato. Foi realizado o Western blotting conforme previamente descrito.^15^ Os anticorpos (p38, p-p38, α-SMA, PCNA e β-actin) foram adquiridos da R&D Systems (Bio-Techne, Minnesota, EUA).

### Análise Estatística

Foram realizadas as análises estatísticas utilizando SPSS 17.0. Os dados são expressos como média ± desvio padrão. Considerando que os dados apresentavam distribuição normal, foram realizadas as comparações entre múltiplos grupos por análise de variância de fator único e as comparações entre dois pelo teste da diferença mínima significativa de Fisher. Foram considerados estatisticamente significativos os valores de p < 0,05.

## Resultados

Os ratos sobreviveram bem 4 semanas após a operação

**Figura 1:**
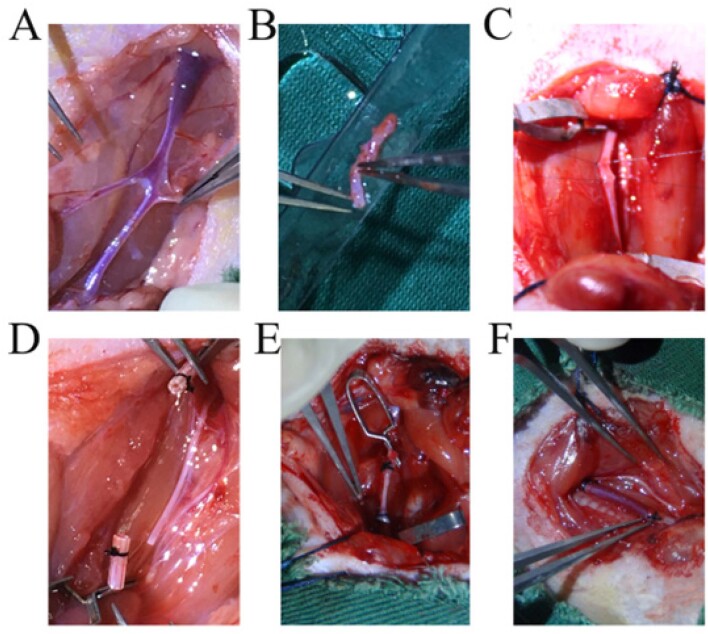
Processo de construção do modelo. Procedimento operacional: Foi utilizado hidrato de cloral 10% para anestesiar os ratos via injeção intraperitoneal. Foi injetada heparina (700 UI/kg) na veia caudal para induzir a heparinização. Foi feita uma incisão vertical de aproximadamente 1 cm no meio do pescoço (desviada para o lado da operação) e as veias foram dissociadas de um lado (A e B). Epitheca de 1 a 2 mm foram obtidas com uma agulha de punção arterial 20G vermelha (BD Company), usada como cânula. A artéria carótida foi isolada até os ramos. Em seguida, duas linhas de sutura e hemoclips foram colocados em ambas extremidades da artéria para bloquear o fluxo sanguíneo (C). O meio da artéria foi isolado e cuidadosamente virado para 1 a 1,2 mm acima da cânula para trazer a íntima para fora (D). Foi usada uma sutura de seda 6/0 para nó e fixação; a veia estava subsequentemente entre artérias isoladas e foi possível abrir os grampos vasculares (E e F). A incisão foi suturada depois que verificamos que o pulso no enxerto de veia estava normal e que não houve sangramento.

Para simular as alterações fisiopatológicas da CRM, nós utilizamos o método de *cuff* aperfeiçoado para construir modelos de enxerto de veia jugular na artéria carótida em um lado; após o enxerto, as veias transplantadas estavam bem preenchidas e os vasos sanguíneos tinham bom pulso (Figura 1). Foram diariamente verificados o estado vital dos ratos e a incisão. Todos os ratos sobreviveram e se recuperaram bem, com pulso bom nas veias enxertadas. Todos os ratos foram sacrificados 4 semanas após a operação. Notavelmente, apenas um rato apresentou oclusão venosa no grupo controle e o fluxo sanguíneo nas veias enxertadas estava sem obstrução. Novo tecido de granulação estava presente nas veias do grupo controle, mostrando tubos mais espessos, edema e rigidez leve. Porém, as veias no grupo atorvastatina apresentavam poucos tecidos novos, sem expansão óbvia e eram facilmente separados.

### A Atorvastatina Reduziu o Espessamento Intimal do Enxerto de Veia

**Figura 2:**
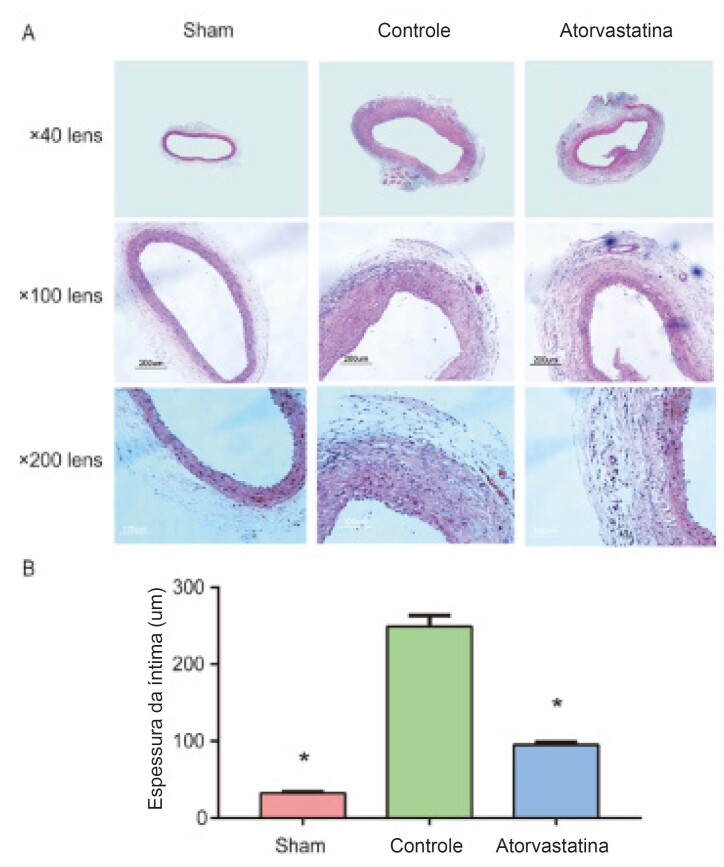
A atorvastatina reduziu o espessamento intimal do enxerto de veia. O tecido dos vasos foi coletado 4 semanas após a operação, fixado com formalina, cortado em seções de tecido de 4 μm e colorido com H&E. As imagens (lente objetivo ×40, ×100 e ×200) foram coletadas e analisadas usando um sistema de aquisição de imagem microscópica Olympus. A íntima do grupo controle foi significativamente mais espessa do que aquela do grupo atorvastatina e do grupo sham (249,3 ± 14,5 versus 95,1 ± 3,6, 32,3 ± 1,7, p < 0,01). *O grupo controle apresentou uma diferença óbvia em relação aos outros dois grupos.

Para avaliar o efeito da atorvastatina na HI, realizamos coloração com hematoxilina-eosina 4 semanas após a cirurgia. Subsequentemente, foi utilizado um sistema computadorizado de análise de imagens para analisar a HI. Os resultados mostraram que a íntima do grupo controle foi significativamente mais espessa do que aquela do grupo atorvastatina e do grupo sham (249,3 ± 14,5 versus 95,1 ± 3,6, 32,3 ± 1,7, p < 0,01; Figura 2A e B). O resultado indicou que a atorvastatina pode inibir a HI no enxerto de veia.

### A Atorvastatina Reduziu a Proliferação Celular na Íntima do Enxerto de Veia

**Figura 3:**
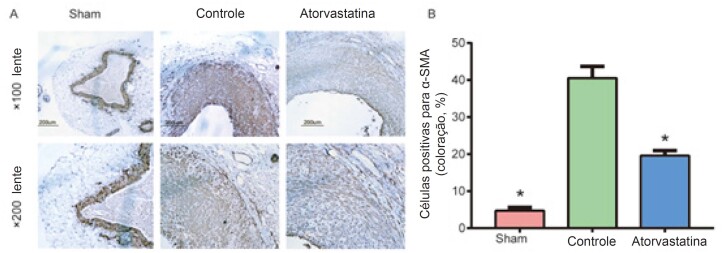
A atorvastatina reduziu a proliferação celular na íntima do enxerto de veia. O tecido dos vasos foi coletado 4 semanas após a operação, fixado com formalina, cortado em seções de tecido de 4 μm e colorido o anticorpo primário anti-α-SMA. As imagens (lente objetivo ×100 e ×200) foram coletadas e analisadas usando um sistema de aquisição de imagem microscópica Olympus. O grupo controle apresentava uma porcentagem de células positivas para α-SMA foi significativamente mais alta do que o grupo atorvastatina e o grupo sham (40,5% ± 3,1% versus 19,6% ± 1,4%, 4,7% ± 0,9%, p < 0,01). *O grupo controle apresentou uma diferença óbvia em relação aos outros dois grupos.

**Figura 4:**
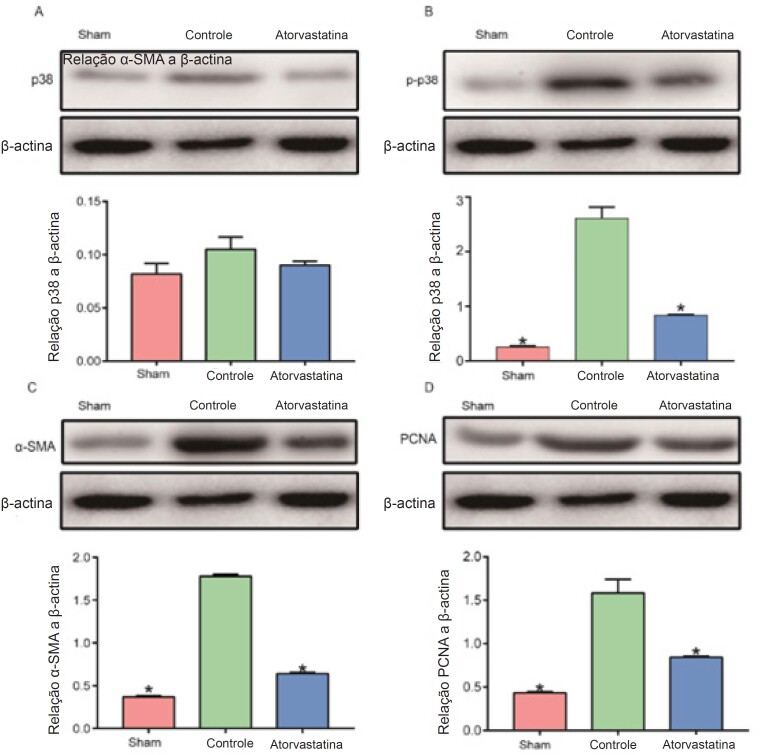
A atorvastatina reduziu a expressão de α-SMA, PCNA e fosforilação de p38 MAPK. O tecido dos vasos coletado 4 semanas após a operação foi colocado em microtubos sem solvente, armazenados a −80 °C e utilizado para detecção de Western blot. A atorvastatina não reduziu o nível de p38 MAPK significativamente (p > 0,05, A). No entanto, inibiu a fosforilação de p38 MAPK (p < 0,01, B) e significativamente reduziu os níveis de α-SMA e PCNA, em comparação com o grupo controle (p < 0,01; C e D). *O grupo controle apresentou uma diferença óbvia em relação aos outros dois grupos.

Realizamos análise imuno-histoquímica de α-SMA e Western blot de α-SMA e PCNA, um indicador do estado de proliferação celular, com a finalidade de investigar os componentes celulares e a proliferação de HI. Além disso, conforme demonstrado na Figura 3, os resultados da coloração imuno-histoquímica de α-SMA mostraram que a porcentagem de células positivas para α-SMA foi significativamente mais alta no grupo controle do que no grupo atorvastatina e no grupo sham (40,5% ± 3,1% versus 19,6% ± 1,4%, 4,7% ± 0,9%, p < 0,01; Figura 3A e B). A atorvastatina significativamente diminuiu os níveis de α-SMA e PCNA, em comparação com o grupo controle (p < 0,01; Figura 4C e D). Estes resultados indicam que as CMLVs são o componente celular principal da HI e que a atorvastatina pode inibir a proliferação das CMLVs e reduzir a HI.

### A Atorvastatina Reduziu a Fosforilação de p38 MAPK

Realizamos Western blot de p38 MAPK e fosforilação de p38 MAPK com a finalidade de investigar a relação entre a atorvastatina e a via p38 MAPK. Os efeitos da atorvastatina em p38 MAPK e na fosforilação de p38 MAPK são apresentados na Figura 4A e B. A atorvastatina inibiu a fosforilação de p38 MAPK (p < 0,01, Figura 4B) mas sem redução significativa de p38 MAPK (p > 0,05, Figura 4A). Estes resultados indicam que a atorvastatina foi capaz de reduzir a HI por meio da inibição da fosforilação de p38 MAPK.

## Discussão

Os resultados deste estudo indicam que a atorvastatina foi capaz de reduzir o acúmulo de CMLVs e inibir a HI suprimindo a via de p38 MAPK. Outros estudos mostraram que, com auxílio da angiotensina II, o tratamento com estatina induziu a fosforilação de p38 MAPK e ERK 1/2 em CMLVs em cultura;^16^ porém, o mecanismo de ação da HI após a CRM ainda não foi esclarecido e não tem sido verificado experimentos em animais. Nos nossos experimentos de reestenose de ponte de veia em ratos, demonstramos que, após o tratamento com atorvastatina, a expressão da proteína de fosforilação de p38 MAPK, α-SMA e PCNA foi reduzida, e houve uma redução significativa na espessura média da HI, bem como uma diminuição significativa na proliferação de α-SMA.

As estatinas têm sido capazes de melhorar os desfechos clínicos dos pacientes com doença cardíaca coronariana, especialmente após intervenção coronariana transluminal percutânea e CRM, devido aos efeitos pleiotrópicos, anti-ateroscleróticos, de inflamação crônica e à inibição da disfunção endotelial.^17^ No entanto, estudos sobre o efeito das estatinas na reestenose do enxerto vascular após CRM são raros. No presente estudo, utilizando coloração com hematoxilina-eosina, verificamos que a atorvastatina foi capaz de inibir a HI de enxertos de veias. Os nossos achados estão de acordo com nosso trabalho anterior, que sugeriu que ratos tratados com sinvastatina apresentavam aumento significativo da área média dos vasos do lúmen em um modelo de acesso vascular em ratos.^18^ Além disso, detectamos a densidade de α-SMA por análise imuno-histoquímica e a expressão de α-SMA e PCNA por Western blot. Estes resultados indicam que a atorvastatina foi capaz de reduzir o acúmulo de CMLVs para inibir HI. Yiguan Xu et al. relataram que a atorvastatina pode inibir neo-HI e promover apoptose de CMLV nas camadas neointimais após injúria da artéria carótida em ratos.^19^ É a primeira vez que observamos o mesmo fenômeno em um modelo de enxerto de veia em rato. Por meio de um mecanismo de ação específico, a atorvastatina levou ao alívio dos danos causados pela injúria do endotélio vascular.

O mecanismo da reestenose inclui trombose, HI e aterosclerose tardia. A proliferação, migração e secreção de células endoteliais e de CMLVs são cruciais à HI, causa principal da reestenose.^20^ Em um estudo prévio, demonstramos que a p38 MAPK é fosforilada em um modelo de enxerto venoso arterializado em ratos, seguido pela ativação da resposta imune inata (inflamação), e um inibidor de p38 MAPK seria capaz de reduzir a proliferação celular induzida por arterialização e diminuir a resposta inflamatória precoce que segue a injúria vascular.^19^ Portanto, testamos a expressão de α-SMA, PCNA, p38 MAPK e fosforilação de p38 MAPK após o tratamento com estatina e os resultados mostraram que a atorvastatina não reduziu o nível de p38 MAPK significativamente (p > 0,05). No entanto, inibiu a fosforilação de p38 MAPK (p < 0,01), e os níveis de α-SMA e PCNA mostraram uma redução significativa, em comparação com o grupo controle (p < 0,01). Conforme relatado por Antonio G. et al. o tratamento com estatina foi capaz de inibir a proliferação das CMLVs em cultura pela via MAPK. No entanto, os experimentos em células que eles realizaram foram apenas *in vivo*, sem a validação de experimentos *in vitro* em animais, e não associaram este mecanismo à HI de reestenose vascular.^16^ O ponto forte principal do nosso estudo foi que realizamos experimentos em ratos, com a construção de um modelo altamente complexo, a fim de verificar se as estatinas foram capazes de reduzir o acúmulo de CMLVs e, ainda, de inibir HI por meio da supressão da via p38 MAPK.

Dessa maneira, os achados de nosso estudo contribuirão para trabalhos clínicos futuros, e deveremos nos concentrar mais na aplicação das estatinas em pacientes submetidos à CRM. Neste estudo, conduzimos apenas experimentos em animais *in vivo*, sem experimentos com células *in vitro*; estudos futuros com investigação mais direta em relação ao mecanismo de ação serão necessários para elucidar o mecanismo de ação no nível molecular. Considerando que a atorvastatina pode reduzir o acúmulo de CMLVs para inibir HI por meio da supressão da via p38 MAPK em modelos de ratos, especulamos que as estatinas também podem ter um efeito preventivo em pacientes após CRM, mas isso ainda precisa ser investigado em estudos futuros. Nossa equipe está atualmente realizando ensaios clínicos controlados sobre os efeitos da aplicação de estatinas na taxa de patência vascular após CRM.

Em conclusão, temos demonstrado que a atorvastatina pode inibir o acúmulo das CMLVs por meio da inibição de via p38 MAPK, levando à inibição da HI. Verificamos este mecanismo pela primeira vez em um modelo de enxerto de veia em ratos. Os resultados desta pesquisa estabelecerão uma base para pesquisa clínica sobre o uso de estatinas na prevenção de reestenose venosa.
