# Supplementary material for: Atorvastatina Reduz o Acúmulo de Células Musculares Lisas Vasculares para Inibir a Hiperplasia Intimal pela Inibição de Via p38 MAPK em um Modelo de Enxerto de Veia em Ratos
Source: Arq Bras Cardiol. 2020 Oct 13;115(4):630–6. [Article in Portuguese] doi: 10.36660/abc.20190231 (PMC8386959; doi:10.36660/abc.20190231)
Supplement: Translation

# Translation: Atorvastatin Reduces Accumulation of Vascular Smooth Muscle Cells to Inhibit Intimal Hyperplasia via p38 MAPK Pathway Inhibition in a Rat Model of Vein Graft

**Authors:** Tianshu Chu, Molin Huang, Zhiwei Zhao, Fei Ling, Jing Cao, Jianjun Ge

**Affiliations:** 1 Hefei China Department of cardiac Surgery, The First Affiliated Hospital of USTC, Division of Life Sciences and Medicine, University of Science and Technology of China, Hefei – China

**Keywords:** Atorvastatin, Myocytes Smooth Muscle, Hyperplasia, Models, Animal, Mitogen Activated Protein Kinases, Myocardial Revascularization, Rats

## Abstract

**Background:**

The rate of saphenous vein graft failure one year after coronary artery bypass grafting ranges from 10% to 25%. The aim of this study was to explore whether atorvastatin can reduce accumulation of vascular smooth muscle cells to inhibit intimal hyperplasia via p38 MAPK pathway inhibition.

**Methods:**

Forty-five Sprague-Dawley rats were randomized to three groups. Thirty rats received a vein graft operation, and they were randomized to be treated with vehicle or atorvastatin; fifteen rats received a sham operation. We detected intimal hyperplasia by hematoxylin-eosin staining and related protein expression by immunohistochemical and Western blot analysis. Comparisons were analyzed by single-factor analysis of variance and Fisher's least significant difference test, with p < 0.05 considered significant.

**Results:**

The intima analyzed by hematoxylin-eosin staining was dramatically thicker in the control group than in the atorvastatin group and sham group (p < 0.01). The outcomes of immunohistochemical staining of α-SMA demonstrated that the percentage of α-SMA-positive cells in the control group was higher than in the atorvastatin group (p < 0.01). We also evaluated α-SMA, PCNA, p38 MAPK, and phosphorylation of p38 MAPK after statin treatment by Western blot analysis, and the results indicated that atorvastatin did not lead to p38 MAPK reduction (p < 0.05); it did, however, result in inhibition of p38 MAPK phosphorylation (p < 0.01), and it significantly reduced α-SMA and PCNA levels, in comparison with the control group (p < 0.01).

**Conclusion:**

We have demonstrated that atorvastatin can inhibit accumulation of vascular smooth muscle cells by inhibiting the p38 MAPK pathway, and it is capable of inhibiting intimal hyperplasia in a rat vein graft model.

## Introduction

Coronary artery disease and related complications are still the main causes of mortality around the world, although there have been many advances in medical therapy. Many studies and clinical guidelines have shown that coronary artery bypass grafting (CABG) surgery reduces morbidity and mortality of patients with three-vessel disease or left main disease, with reduced ejection fraction compared to percutaneous coronary intervention.^1^ Notably, one year after CABG, the rate of saphenous vein graft (SVG) failure can be 10% to 25%, and in 1 to 5 years, the rate will be increased by 1 % to 2% per year.^2^^,^^3^ Moreover, in 6 to 10 years, the failure rate will increase at a rate of 4% to 5% every year, due to atherosclerosis.^4^ The mechanism of SVG restenosis includes thrombosis, intimal hyperplasia (IH) and atherosclerosis. The proliferation and migration of endothelial cells and vascular smooth muscle cells (VSMCs) are crucial to IH, and IH is the main cause of SVG restenosis.^5^ However, the mechanism of IH is not clear, and it is still not known which prevention method and therapy would be most effective.

A substantial amount of evidence suggests that statin treatment reduces cardiovascular risk; therefore, when low-density lipoprotein cholesterol levels are higher than 100 mg/dL, statin therapy is recommended for patients with coronary artery disease.^6^ Many observations have indicated that preoperative statin treatment could reduce morbidity, postoperative mortality, and complications, and recent evidence has indicated that statin treatment after CABG can reduce the rate of SVG disease by inhibiting IH, indicating that optimal statin treatment is crucial to the assessment of the long-term benefit of CABG.^7^^–^^9^ The “response-to-injury” hypothesis proposed by Russell Ross is widely accepted; this hypothesis states that, following arterialization, SVGs instantly undergo injury (e.g. ischemia, hypoxia, shear stress, or surgical trauma), leading to an initiating event in the inflammatory response, followed by morphological and functional changes leading to IH, as the result of accumulation of VSMCs and endothelial cell dysfunction.^10^ The dysfunction, proliferation and migration of these cells are stimulated by phosphorylation of p38 mitogen activated protein kinases (p38 MAPKs), extracellular signal-regulated kinases (ERK), c-Jun and N-terminal kinases.^11^ Studies have demonstrated that inhibition of p38 MAPK could reduce the innate immune response and consequently inhibit IH following SVG arterialization.^12^^,^^13^

Based on these studies on IH, we hypothesized that atorvastatin could reduce the accumulation of VSMCs to inhibit IH by suppressing the p38 MAPK pathway. We verified our hypothesis by using a rat vein graft model with statin treatment to detect IH by hematoxylin-eosin staining and correlated protein expression by immunohistochemical and Western blot analysis. We found that atorvastatin was able to inhibit phosphorylation of p38 MAPK to reduce accumulation of VSMCs and, furthermore, inhibit IH.

## Materials and Methods

### Experimental Animals and Surgical Procedure

All animal experiments in this study were performed according to protocols approved by the Institutional Committee for Use and Care of Laboratory Animals. Forty-five male, pathogen-free, 8- to 10-week-old Sprague-Dawley rats, weighing 200 to 220 g were provided by the Anhui Lab Animal Research Center and identified by the Medical Ethics Committee of Anhui Medical University. They were randomized (completely randomized design) to 3 groups, containing 15 rats each, and fed for 4 weeks after operation. Thirty rats received a vein graft as described previously;^14^ the method was used to construct rat models of right jugular vein grafts on common carotid artery, and the rats were randomized to be treated with vehicle (control group, administered with distilled water continuously by gavage for 4 weeks) or atorvastatin (atorvastatin group, 15 mg/kg, dissolved in distilled water). Fifteen rats received a sham operation (sham group), defined as simulation of the operation process, without venous arterialization and medical intervention.

### Sample Collection

We collected each rat's vein graft at the fourth week after operation. Fully anaesthetized rats were fixed on the operating table, heparinized as before and operated in the same way, through the same approach. For histological analysis, vein grafts were placed in microtubes with paraformaldehyde, and fixed at 4 °C for 24 hours. Vein grafts with Western-Blot were placed in solvent-free microtubes and then stored at −80 °C. Rats were euthanized by cervical dislocation and handled properly.

### Histologic and Immunohistochemical Analysis

Morphometric analysis of intima was performed by hematoxylin-eosin staining, using a hematoxylin and eosin staining kit (Beyotime Biotechnology, Shanghai, China). An Olympus microscope image acquisition system was used to collect images of sections (×40, ×100, and ×200 objective lens) and measure intimal thickness. Two independent researchers performed the measurements and data analysis. We selected sections of grafted veins; subsequently, we measured 16 points of intimal thickness and calculated the mean. Tissue sections were tested for cell proliferation using an immunohistochemistry analysis kit for α-smooth muscle actin (α-SMA) (R&D Systems, Bio-Techne, Minnesota, USA), the specific protein of VSMCs. All images (×100 and ×200 objective lens) were captured using an Olympus microscope image acquisition system (Olympus, Japan) and processed with Image-J 1.48u software (National Institutes of Health, Bethesda, USA). A total of 10 observation views were applied to calculate the average percentage of α-SMA-positive cells for each rat.

### Western Blot Analysis

Four weeks after the operation, equivalent amounts of vein graft proteins from the three groups were electrophoresed in sodium dodecyl sulfate/10% polyacrylamide gel and blotted onto PVDF membranes (Sigma-Aldrich, USA). The membranes were subsequently incubated with anti-phospho-specific p38 MAPK antibodies, anti-non-phosphorylated p38 MAPK antibodies, anti-non-phosphorylated α-SMA antibodies, and anti-non-phosphorylated proliferating cell nuclear antigen (PCNA) antibodies, followed by incubation with an anti-mouse IgG-peroxidase. Western blotting was performed as previously described.^15^ The antibodies (p38, p-p38, α-SMA, PCNA and β-actin) were acquired from R&D Systems (Bio-Techne, Minnesota, USA).

### Statistical Analysis

Statistical analyses were performed using SPSS 17.0. Data are shown as mean ± standard deviation. As the data showed normal distribution, comparisons between multiple groups were analyzed by single-factor analysis of variance (ANOVA), and comparisons between two groups were made by Fisher's least significant difference test. P values < 0.05 were considered statistically significant.

## Results

### Rats Survived Well 4 Weeks After Operation

To simulate the pathophysiological changes of CABG, we used the improved cuff method to construct rat models of jugular vein graft on carotid artery in one side; after grafting, the transplanted veins were well filled, and the blood vessels had good pulse (Figure 1). Rats’ vital status and incision were checked every day, and all rats survived and recovered well with good pulse in the grafted veins. All rats were euthanized 4 weeks after operation. Notably, only one rat had venous occlusion in the control group, and blood flow in other grafted veins was unobstructed. New granulation tissue was present in the veins of the control group, showing thickened tubes, edema, and mild stiffness. However, veins in the atorvastatin group had few fresh tissues with no obvious expansion, and they were easily separated.

**Figure 1:** Model construction process. Operating procedure: 10% chloral hydrate was used to anaesthetize rats via intraperitoneal injection. Heparin (700 IU/kg) was injected through the caudal vein to induce heparinization. A vertical incision of approximately 1 cm was made in the middle of the neck (deflected to the operation side) and the veins were dissociated on one side (A and B). Epitheca of 1 to 2 mm were taken using 20G red arterial puncture needle (BD Company), used as cannula. The carotid artery was isolated until the branches. Then, two suture traction lines and hemoclips were placed at both ends of the artery to block blood flow (C). The middle of the artery was isolated and turned carefully to 1 to 1.2mm above the cannula to bring intima outside (D). 6/0 silk suture was used for knot and fixation; the vein was subsequently between isolated arteries, and we were able to open vascular clamps (E and F). The incision was sutured after we had verified that pulse in the vein graft was normal and that there was no bleeding.

### Atorvastatin Reduced Intimal Thickening of the Vein Graft

To evaluate the effect of atorvastatin on IH, we performed hematoxylin-eosin staining 4 weeks after surgery. Subsequently, a computer image analysis system was used to analyze IH. The results showed that the intima of the control group was significantly thicker than that of the atorvastatin group and the sham group (249.3 ± 14.5 versus 95.1 ± 3.6, 32.3 ± 1.7, p < 0.01; Figure 2A and 2B). The result indicated that atorvastatin is able to inhibit IH in the vein graft.

**Figure 2:** Atorvastatin reduced intimal thickening of the vein graft. The vessel tissue was collected 4 weeks after operation, fixed with formalin, sliced to 4 μm tissue sections, and stained with H&E. Images (×40, ×100, and ×200 objective lens) were collected and analyzed on an Olympus microscope imaging system. The intima of the control group was significantly thicker than that of atorvastatin group and the sham group (249.3 ± 14.5 versus 95.1 ± 3.6, 32.3 ± 1.7, p < 0.01). *The control group had an obvious difference with the other two groups.

### Atorvastatin Reduced Cell Proliferation in Intimal of The Vein Graft

We performed immunohistochemistry analysis of α-SMA and Western blot of α-SMA and PCNA, an indicator of cell proliferation status, in order to explore the cellular components and proliferation of IH. Furthermore, as shown in Figure 3, the results of immunohistochemical staining of α-SMA showed that the percentage of α-SMA-positive cells was significantly higher in the control group than in the atorvastatin group and the sham group (40.5% ± 3.1% versus 19.6% ± 1.4%, 4.7% ± 0.9%, p < 0.01; Figure 3A and B). Atorvastatin significantly decreased α-SMA and PCNA levels, in comparison with control group (p < 0.01; Figure 4C and D). These results indicate that VSMCs are the main cellular component of IH and that atorvastatin can inhibit the proliferation of VSMCs and reduce IH.

**Figure 3:** Atorvastatin decreased cell proliferating of the vein graft. The vessel tissue was collected 4 weeks after operation, fixed with formalin, sliced to 4 μm tissue sections, and stained with the primary antibody anti-α-SMA. Images (×100 and ×200 objective lens) were collected and analyzed on an Olympus microscope imaging system. The control group had a significantly higher percentage of α-SMA-positive cells than the atorvastatin group and the sham group (40.5% ± 3.1% versus 19.6% ± 1.4%, 4.7% ± 0.9%, p < 0.01). *The control group had an obvious difference the other two groups.

**Figure 4:** Atorvastatin reduced the expression of α-SMA, PCNA and phosphorylation of p38 MAPK. The vessel tissue collected 4 weeks after operation was placed in solvent-free microtubes, stored at −80 °C, and used for Western blot detection. Atorvastatin did not significantly reduce the level of p38 MAPK (p > 0.05, A). However, it inhibited phosphorylation of p38 MAPK (p < 0.01, B) and significantly reduced α-SMA and PCNA levels, in comparison with the control group (p < 0.01; C and D). *The control group had an obvious difference the other two groups.

### Atorvastatin Reduced Phosphorylation of p38 MAPK

We performed Western blot of p38 MAPK and phosphorylation of p38 MAPK in order to explore the relation between atorvastatin and the p38 MAPK pathway. The effects of atorvastatin on p38 MAPK and phosphorylation of p38 MAPK are shown in Figure 4A and B. Atorvastatin inhibited phosphorylation of p38 MAPK (p < 0.01, Figure 4B) but without significantly reducing p38 MAPK (p > 0.05, Figure 4A). These results indicate that atorvastatin was able to reduce IH by inhibiting phosphorylation of p38 MAPK.

## Discussion

The results of this study indicate that atorvastatin was able to reduce the accumulation of VSMCs and inhibit IH by suppressing the p38 MAPK pathway. Other studies have shown that, with the help of angiotensin II, statin treatment induced phosphorylation of p38 MAPK and ERK 1/2 in cultured VSMCs;^16^ however, the action mechanism of IH after coronary artery bypass has not been made clear, and it has not been verified in animal experiments. In our experiments of vein bridge restenosis in rats, we demonstrated that, after atorvastatin treatment, the protein expression of phosphorylation of p38 MAPK, α-SMA, and PCNA was reduced, and there was a significant reduction in the average thickness of IH, as well as a significant decrease in proliferation of α-SMA.

Statins have been able to improve clinical outcomes of patients with coronary heart disease, especially after percutaneous transluminal coronary intervention and CABG, due to pleiotropic, anti-atherosclerotic, and chronic inflammation effects, as well as inhibition of endothelial dysfunction.^17^ However, studies about the effect of statins on vascular graft restenosis after CABG are rare. In the current study, using hematoxylin-eosin staining, we found that atorvastatin was able to inhibit IH of vein grafts. Our findings are in agreement with our previous work, which has suggested that rats treated with simvastatin showed significant growth in the mean lumen vessel area in a rat model of vascular access.^18^ Furthermore, we detected α-SMA density by immunohistochemistry and the expression of α-SMA and PCNA by Western blot. These results indicated that atorvastatin was able to reduce accumulation of VSMCs to inhibit IH. Yiguan Xu et al. reported that atorvastatin can inhibit neo-IH and promote VSMC apoptosis in neointimal layers after carotid artery injury in rats.^19^ This is the first time that we have observed the same phenomenon in a rat model of vein graft; namely, through a specific action mechanism, atorvastatin led to alleviation of the damage caused by vascular endothelial injury.

The mechanism of restenosis includes thrombosis, IH, and late atherosclerosis. Proliferation, migration, and secretion of endothelial cells and VSMCs are crucial to IH, the leading cause of restenosis.^20^ In a previous study, we showed that p38 MAPK is phosphorylated in a model of arterialized vein grafts followed by activation of the innate immune response (inflammation), and a p38 MAPK inhibitor could reduce arterialization-induced cell proliferation and downregulate the early inflammatory response that follows vascular injury.^19^ Hence, we tested the expression of α-SMA, PCNA, p38 MAPK, and phosphorylation of p38 MAPK after statin treatment, and the results showed that atorvastatin did not significantly reduce the level of p38 MAPK (p > 0.05). However, it inhibited phosphorylation of p38 MAPK (p < 0.01), and the α-SMA and PCNA levels showed a significant decrease, in comparison with the control group (p < 0.01). As reported by Antonio G. et al, statin treatment was able to inhibit proliferation of VSMCs in culture via the MAPK pathway. However, the cell experiments they completed were only *in vivo*, without validation of *in vitro* animal experiments, and they did not link this mechanism to IH of vascular restenosis.^16^ The main strength of our study is that we performed our experiments in rats, with the construction of a highly complex model, in order to verify that statins were able to reduce accumulation of VSMCs and, furthermore, inhibit IH by suppressing p38 MAPK pathway.

Therefore, the findings from our study will contribute to future clinical work, and we should focus more on the application of statins in patients with CABG. In this study, we conducted only animal experiments *in vivo*, without *in vitro* cell experiments; future studies with more direct action mechanism research are needed to elucidate the action mechanism at the molecular level. As atorvastatin could reduce the accumulation of VSMCs to inhibit IH by suppressing the p38 MAPK pathway in rat models, we speculate that statins may also have a preventive effect in patients after CABG, but this still needs to be investigated in future studies. Our team is currently carrying out controlled clinical trials on the effects of statin application on the vascular patency rate after CABG.

In conclusion, we have demonstrated that atorvastatin can inhibit the accumulation of VSMCs by inhibiting the p38 MAPK pathway, leading to IH inhibition. We have verified this mechanism for the first time in a rat model of vein graft. These research results will lay a foundation for basic and clinical research on statin use for prevention of venous restenosis.
